# *Alcyonium* Octocorals: Potential Source of Diverse Bioactive Terpenoids

**DOI:** 10.3390/molecules24071370

**Published:** 2019-04-08

**Authors:** Ahmed Abdel-Lateff, Walied Mohamed Alarif, Najla Ali Alburae, Mardi Mohamed Algandaby

**Affiliations:** 1 Department of Natural Products and Alternative Medicine, Faculty of Pharmacy, King Abdulaziz University, P.O. Box 80260, Jeddah 21589, Saudi Arabia; 2 Department of Pharmacognosy, Faculty of Pharmacy, Minia University, Minia 61519, Egypt; 3 Department of Marine Chemistry, Faculty of Marine Sciences, King Abdulaziz University, P.O. Box 80207, Jeddah 21589, Saudi Arabia; 4 Department of Biology, Faculty of Science, King Abdulaziz University, P.O. Box 80203, Jeddah 21589, Saudi Arabia; nalbourai@kau.edu.sa or nalbourae@gmail.com (N.A.A.); malgandaby@yahoo.com (M.M.A.); 5 Biology Department, College of Science, Princess Nourah bint Abdulrahman University, P.O. Box 84428, Riyadh 11671, Saudi Arabia

**Keywords:** Alcyoniidae, mevalonates, steroids, anti-inflammatory, antimicrobial, antifeedant

## Abstract

*Alcyonium* corals are benthic animals, which live in different climatic areas, including temperate, Antarctic and sub-Antarctic waters. They were found to produce different chemical substances with molecular diversity and unique architectures. These metabolites embrace several terpenoidal classes with different functionalities. This wide array of structures supports the productivity of genus *Alcyonium*. Yet, majority of the reported compounds are still biologically unscreened and require substantial efforts to explore their importance. This review is an entryway to push forward the bio-investigation of this genus. It covers the era from the beginning of reporting metabolites from *Alcyonium* up to March 2019. Ninety-two metabolites are presented; forty-two sesquiterpenes, twenty-five diterpenes and twenty-five steroids have been reported from sixteen species.

## 1. Introduction

The marine environment is represented by two-thirds of the earth and epitomizes harsh parameters. It has a wide range of temperature; ranged from −1.5 °C to 350 °C, pressure ranged from 1 to over 1000 atmosphere, light ranged from complete darkness to extensive photic zones and nutritional-rich till nutrient-spar [1,2,3]. 

Thirty-four animal phyla were taxonomically identified, however, thirty-six were found in a marine habitat. The marine species counts around 240,000 known species, albeit less than five percent of the deep sea has been explored [4,5]. Production of the unique metabolites from marine organisms could be explained by the harsh and competitive conditions. Although the terrestrial sources are providing unique bioactive metabolites, the marine organisms produce a considerable number of unprecedented bioactive substances, which have a great possibility to be a lead drug [6]. Blunt and his co-workers reported that the identified marine metabolites estimated to be 31,000 (i.e., 1000 substances per year) [7]. The molecular structures associated, particularly, produced from marine organisms, are varying from low molecular weight to complex form [8,9,10,11,12,13,14]. These metabolites enhance marine invertebrate’s survival by providing chemical defense. They play a crucial role in the adaptation of the marine organisms to the physical and chemical extreme conditions. The marine metabolites interfere with receptors and enzymes of coexisting marine competitors and predators. This emphasized the hypothesis that several of those compounds could interfere with molecular targets [15,16,17,18,19]. 

Alcyonacea (Phylum, Cnidaria; class, Anthozoa; subclass, Octocorallia; order, Alcyonacea) constitutes an important group of marine invertebrates, widely distributed in the coral reefs. They are quite numerous throughout the tropical waters, mainly live in the intertidal zones on inner reefs below the stony corals [14]. They are less prone to damage or ailments from collecting and shipping than the stony corals. They protect themselves by the production of certain chemical mediators, due to the absence of skeletal defenses [3,14]. 

Soft corals have proven to be a biochemical warehouse for production of bioactive terpenoidal metabolites particularly, those belonging to the family, Alcyoniidae (37 genera) [3,20]. These types of metabolites show roles in protection and taxonomical identification (i.e., markers) [21]. Genus *Alcyonium* (Flame corals) are small soft corals. They live in colonies of polyps (round body) and forming erect fleshy masses. The absence of an internal skeleton was observed. Each polyp contains eight small, feathery tentacles called pinnates. These pinnates contain stinging cells that they utilized to catch their prey. They are micro carnivores that feed on planktonic animals. Their body is a pale yellow color, however, the stem of the polyp is orange and the polyps are bright red. Genus *Alcyonium* comprises 141 species, of which 71 were accepted to be transferred to other genera [4,5,8].

In the current review, the isoprenoidal derivatives which, isolated from genus *Alcyonium*, are presented. These compounds showed certain effects on some diseases and could have a coming role in drug discovery. It is interesting to discuss the future perspectives of the chemical structures and possible biological activity relationships. Sixteen *Alcyonium* species of different geographical locations have been chemically investigated, resulted in the identification of ninety-two metabolites which categorized under three classes; sesquiterpenes, diterpenes and steroids. Extensive literature surveys were performed employing different scientific databases (e.g., SciFinder, Scopus, PubMed, Scholar, ScienceDirect and Web of Science), indicated the scarce or almost absence of review interested in this theme. 

## 2. Terpenoids from *Alcyonium*

**Figure 1:**
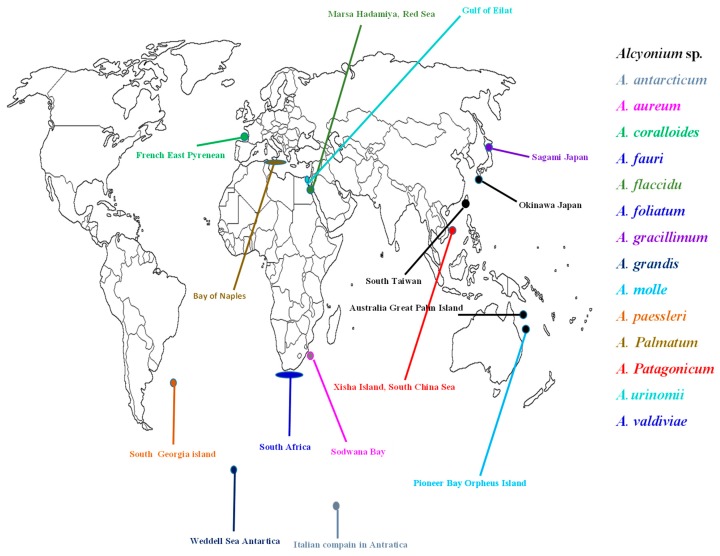
Locations of the investigated *Alcyonium* species.

**Figure 2:**
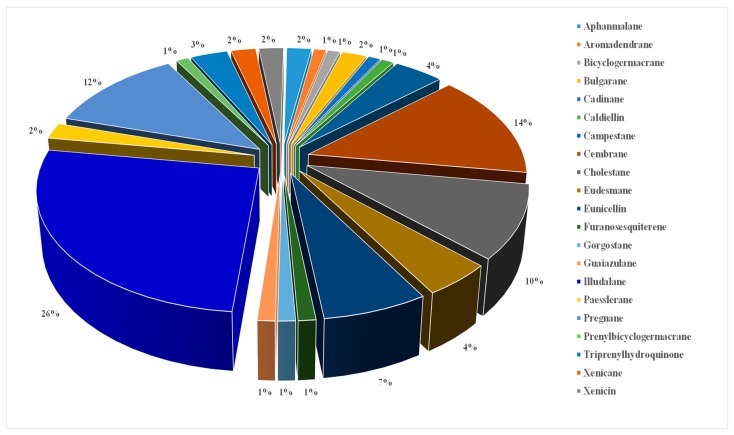
Percentage of chemical classes of *Alcynocium* terpenoids.

Soft corals of the genus *Alcyonium* are widely spread all over the oceans. Some of them were chemically and biologically investigated (Table 1, Figure 1). Up to March 2019, ninety-two terpenoidal derivatives have been isolated and identified from sixteen species of genus *Alcyonium*, namely, *Alcyonium* sp., *A. antarcticum*, *A. coralloides*, *A. fauri*, *A. flaccidu*, *A. foliatum*, *A. gracillimum*, *A. grandis*, *A. molle*, *A. paessleri*, *A. palmatum*, *A. patagonicum*, *A. utinomii*, and *A. valdiviae* (Table 1 and Figure 2). 

**Figure 3:**
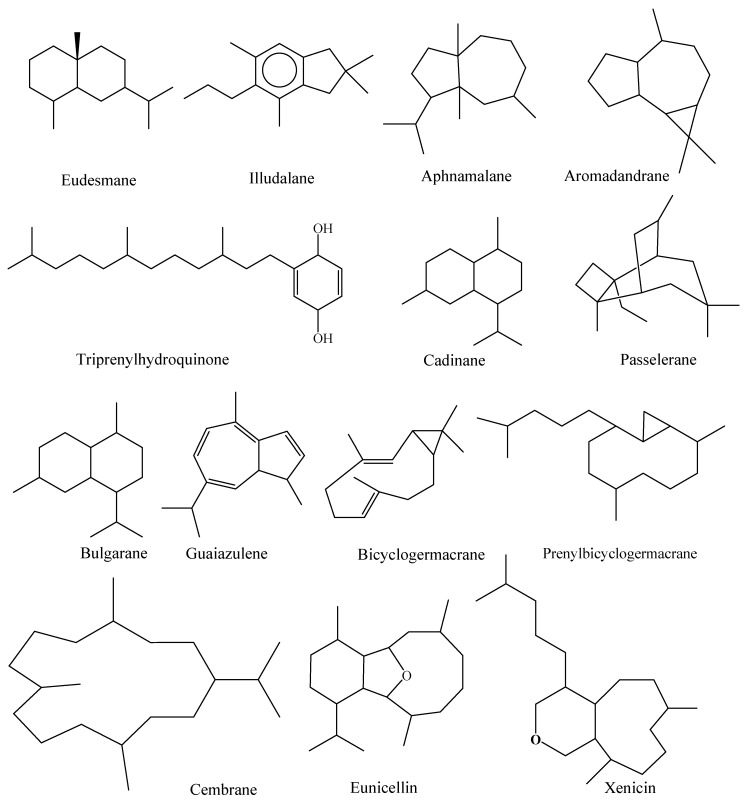
Selected chemical structures of *Alcyonium* terpenoidal classes.

Eighty-six isoprenoids have been recorded from *Alcyonium* for the first time, and the remaining six were previously reported from other marine sources. Sesquiterpenoids are eminent metabolites from *Alcyonium*, which were categorized under eleven carbo-skeleton types; aphanmalane, aromadendrane, bulgarane, cadinane, bicyclogermacrane, eudesmane, furanosesquiterene, guaiazulene illudalane, paesslerane, and triprenylhydroquinone (Figure 2 and Figure 3). Besides, diterpenoids from *Alcyonium* are classified into six classes, cembrane, cladiellin, eunicellin, prenylbicyclogermacrane, xenicin, and xenicane. Finally, twenty-five steroids have been identified. Interestingly, steroids cholestane (C-27), campestane (C-28), gorgostane (C-30) along with pregnane (C21) carbon skeleton were all identified. The diversity of the terpenoidal content of genus *Alcyonium* is a source of 92 metabolites which categorized under 21 classes. This addressed that, the metabolites were obtained from 20% of the identified species, thus, the chem-biological investigations of the rest (80%) are urgently required. 

### 2.1. Sesquiterpenes

**Figure 4:**
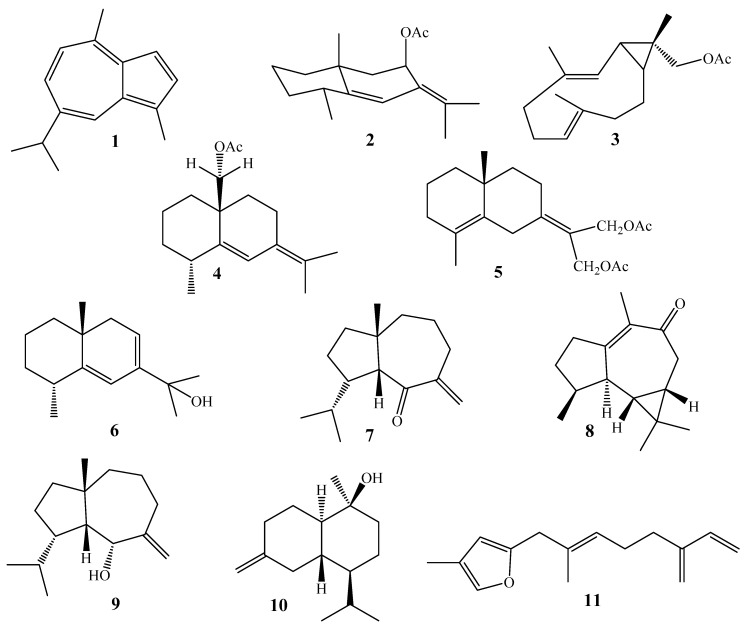
Chemical structures of compounds **1**–**11**.

The chemical diversity of the aforementioned sesquiterpenoidal classes emphases the importance of genus *Alcyonium* as a potential source of novel metabolites. A bicyclic sesquiterpenoidal, guaiazulene (**1**), a pigment obtained from *Alcyonium* sp., which was collected from the North East Bay, Great Palm Island of Australia. It was used as a taxonomical marker for the gorgonian soft coral [22]. Chemical investigation of the Mediterranean *A. coralloides*, collected from the French East Pyrenean, yielded two novel sesquiterpenes (+)-coralloidin-A (**2**), and (−)-coralloidin-B (**3**) (Figure 4 and Table 1) [23]. Novel eudesmane sesquiterpenes, coralloidin C, D and E (**4**–**6**) have been identified from the same species. The absolute stereochemistry of **4** was estimated by application of the exciton-coupling method and confirmed by interpretation of the negative and positive cotton effects after measuring the Circular Dichroism spectra [24]. 

A south African nudibranch, *Leminda millecra*, was investigated chemically and led to the isolation of four novel aromadendrane and aphnamalane, namely, millecrone A and B (**7** and **8**), and millecrol A and B (**9** and **10**). It was surprising that the same metabolites were obtained from the organic extract of spicules in the dissected digestive glands of the soft corals *A. foliatum* and *A. valdiviae* [25,26]. Although millecrone B (**8**) was inactive against the growth of *Candida albicans* mellicrone A showed inhibition at 50 g/disk; while millecrol A and B (**9** and **10**) showed antimicrobial activity against *Staphylococcus aureus* and *Bacillus subtilis* [25,26].

As known, furanosesquiterpenoid (**11**) has been identified from the bay of Naples octocoral *A. palmatum.* This compound and its congeners (e.g., 2,4-disubstituted furanosesquiterpene) play a role in the taxonomy of the Alcyonacea order [27]. 

*A. fauri* is an endemic southern Africa soft coral, has been investigated and yielded three sesquiterpene hydroquinones, rietone (**12**), 8′-acetoxyrietone (**13**) and 8′-desoxyrietone (**14**) [28]. The NCI’s CEM-SS cell line assay was designed to evaluate the metabolites which have effect at any stage of HIV virus reproductive cycle and fortunately, rietone (**12**) showed moderate effect. It was remarkable that *A. fauri* collected during this study was found growing on living *Hadromerida* sponges (*Tethya* species) and certain study has been done indicated that there is no chemical affinities or similarity between *A. fauri* and sponge or other soft coral, regarding the production of metabolites [28].

**Figure 5:**
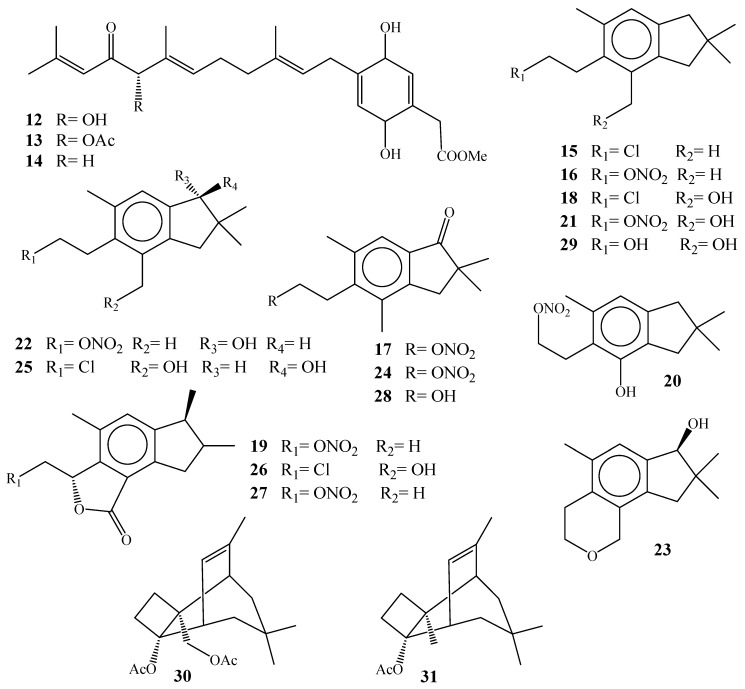
Chemical structures of compounds **12**–**31**.

Fifteen rare illudalane sesquiterpenes (Figure 5); alcyopterosins A-O (**15**–**29**) had been isolated from sub-Antarctic soft coral *A. paessleri*, collected from the South Georogia Islands, eight out of fifteen compounds have a nitrate ester group (**16**, **17**, **19**–**22**, **24** and **27**), while four compounds are chlorinated (**15**, **18**, **26** and **26**) [29]. These metabolites were the first illudalane sesquiterpenoidal derivatives, which were reported from marine organisms. The stereochemistry of the alcyopterosins showed a different configuration of the hydroxylated position (C-10). Compound **22** was levorotatory while **23** and **27** were dextrorotatory. The absolute stereochemistry was established by the implementation of the modified Mosher method led to the establishment of the chemical structures of **23**, **26**, and **27** had 10*S* configuration, while **22** was 10*R*. Compound **19** showed mild cytotoxicity against Hep-2 (human larynx carcinoma) cell line (IC_50_ 13.5 µM), while compounds **15**, **17**, and **22** were cytotoxic against HT-29 (human colon carcinoma) at 10 µg/mL. Further investigation of the same USA group and marine organisms led to the identification of two novel tricyclic sesquiterpenoids, paesslerins A and B (**30**–**31**) [30]. 

**Figure 6:**
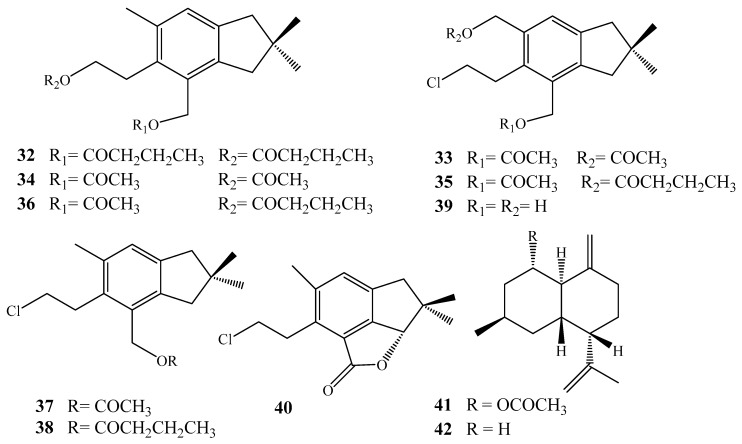
Chemical structures of compounds **31**–**42**.

Lipophilic extract of the Antartic *A. grandis*, collected from Weddell Sea, Antartica yielded nine unreported sesquiterpenoids, 4,12-*bis*-*n*-butanoylalcyopterosin O (**32**), 13-acetoxy-12-acetyl alcyopterosin D (**33**) (Figure 6), 4,12-*bis*(acetyl) alcyopterosin O (**34**), 12-acetyl-13-*n*-butanoxy alcyopterosin D (**35**), 12-acetyl-4-*n*-butanoylalcyopterosin O (**36**), 12-acetylalcyopterosin D (**37**), 12-*n*-butanoylalcyopterosin D (**38**), 13-hydroxy alcyopterosin (**39**) and alcyopterosin P (**40**). The lipophilic extract exhibited a feeding-deterrent effect towards the Antarctic predator *Odontaster validus* and proved to have a potent repellent effect [31]. 

*A. antarcticum*, collected during the XVII Italian campaign in Antarctica off Terra Nova Bay [31], yielded a rare bulgarane sesquiterpene; alcyonicene (**41**), deacetoxyalcyonicene (**42**), and 4-methyl-2-[(*E*)-2-methyl-6-methyleneocta-2,7-dienyl]-furan (**11**) [32]. Feeding-deterrence and ichthyotoxic effects of alcyonicene (**41**), as well as 4-methyl-2-[(*E*)-2-methyl-6-methyleneocta-2,7-dienyl]-furan were preliminarily evaluated by conducting assays with *Carassius auratus* and *Gambusia affinis* [32].

### 2.2. Diterpenes

**Figure 7:**
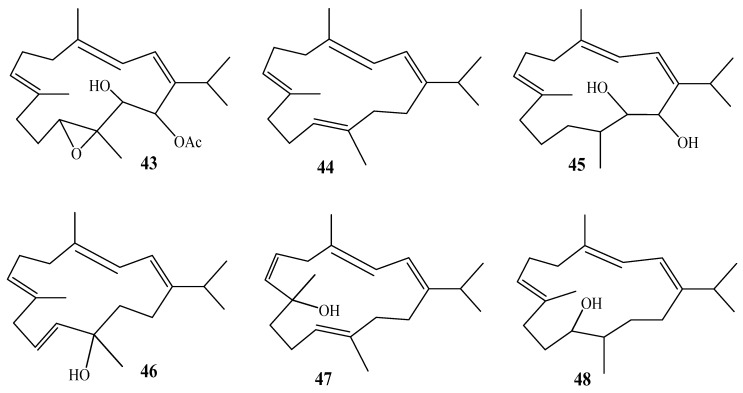
Chemical structures of compounds **43**–**48**.

A cembranoid-type diterpene, 11,12-epoxy-13-hydroxy-14-acetoxycembrene-C (Flaccidoxide, **43**) (Figure 7), was reported for the first time from *A. flaccidum*, along with known cembranoids, cembrene-C (**44**) and sarcophytol-B (**45**). This species was collected from Marsa-Hadamiya (Gulf of Suez, Red Sea) [33]. 

Examination of *A. utinomii*, was collected from the Gulf of Suze, led to the isolation of three cembranoidal derivatives with the same molecular weight, alcyonol-A (**46**), alcyonol-B (**47**), and alcyonol-C (**48**). The difference between the chemical structure of compounds **46** and **47** is mainly in the location of the hydroxyl group [34]. 

**Figure 8:**
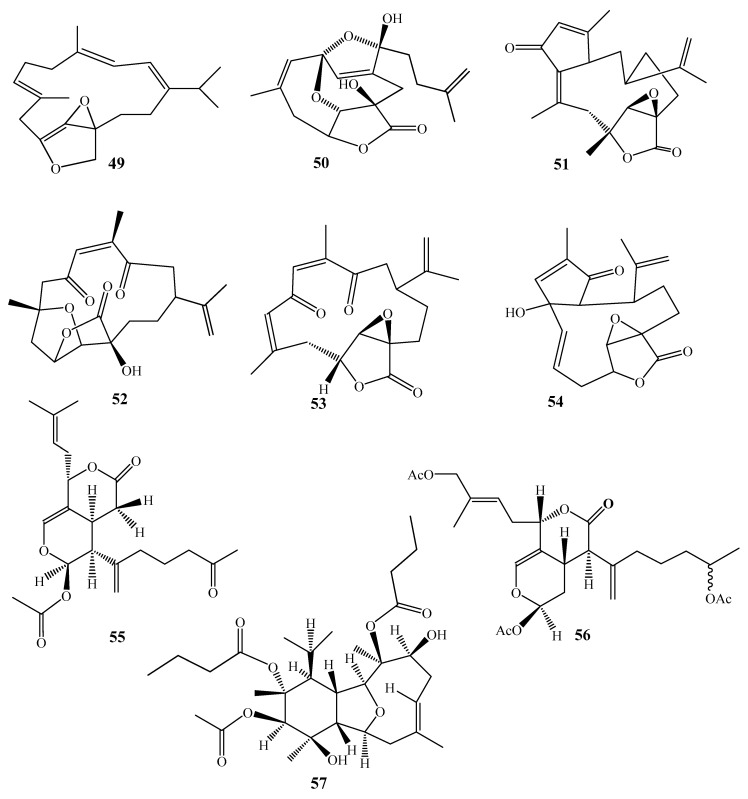
Chemical structures of compounds **49**–**57**.

D’ Ambrosio et al. reported two new cembranoidal metabolites, coralloidolide A (**49**) & B (**50**) with peculiar structure from the French East Pyrenean of the Mediterranean Sea *A. coralloides* (Figure 8). The two structures are peculiar with a rare (7*Z*)-configuration. This feature is rare in cembranoids [35]. A study from the same group reported three novel metabolites; 3,7-cyclized cembranoid (Coralloidolide C, **51**), O-bridged diketonic cembranolide (Coralloidolide D, **52**) and diketonic epoxycembranolide (coralloidolide E, **53**) [36]. Further investigation of the same species and same group led to reporting of the first example of 2, 6-cyclized cembranolide (Coralloidolide F, **54**) [37]. 

Alcyonolide (**55**) is an unusual diterpenoidal acetate, was isolated from an Okinawan soft coral *Alcyonium* sp. [38]. Alcyonolide-5 (**56**) is a triacetate derivative, obtained from *Alcyonium* sp. collected from Lamont Reef in the Capricorn Bunker group [39]. These metabolites were believed to be derived from a xenicin-type precursor.

A cladiellin-based diterpene (1*S*,2*R*,3*S*,4*R*,5*R*,6*S*,8*E*,11*S*,12*R*,13*S*,14*S*)-3-acetoxy-2,12–dibutanoyl oxycladiell-8-ene-4,11-diol (**57**) has been reported from *A. molle*, collected at Pioneer Bay, Orpheus Island. Its absolute configuration was based on the kinetic resolution method of Horeau [40]. 

**Figure 9:**
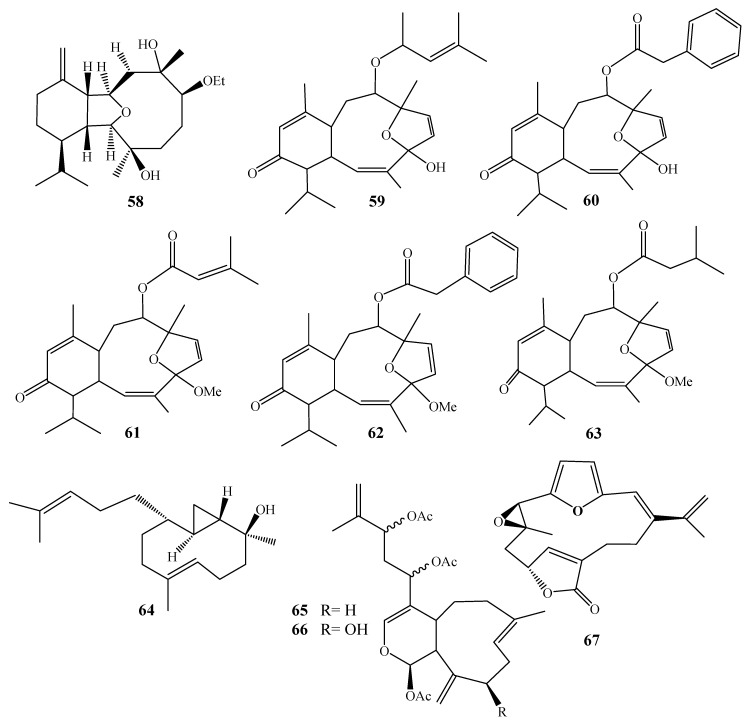
Chemical structures of compounds **58**–**67**.

Patagonicol (**58**) (Figure 9), a new diterpene of eunicellin skeleton has been reported from the Soft coral *A. patagonicum* collected from the Xisha islands off the south China Sea. Its structure was confirmed by X-ray diffraction [41]. 

The soft coral *A. valdivae*, collected from Coffee Bay, Transkei, South Africa, yielded five diterpene esters, valdivone A (**59**), valdivone B (**60**), 4-*O*-methyl valdivone A (**61**), 4-*O*-methyl valdivone B (**62**) and dihydrovaldivone A (**63**). Carbon skeleton of valdivones is eunicellin-type which closely related to sarcodictyins. The difference between them is the location of the ether ring however they produced by different soft corals (i.e., *A. valdivae* (order Alcyonacea) and *Sarcodictyon roseum* (order Stolonifera)). Valdivones A (**59**) and B (**60**) show strong inhibition of chemically-induced inflammation in the mouse ear assay, however, no inhibition on the bee venom phospholipase A. Finally, the valdivones showed no effect against a standard panel of bacteria and fungi [42]. 

A diterpene of the prenylbicyclogermacrane skeleton wasn’t widely occurred among marine organisms. Fortunately, *A. palmatum was* collected from Mazara de1 Vallo (West Sicily), led to the isolation of palmatol (**64**). Palmatol showed toxicity against *Gumbosia offinis* as well as cytotoxic against brine shrimp (*Artemia salina)* [43].

Xenicane-type diterpenoid was reported from *Alcyonium*, for instance, zahavin A (**65**), and zahavin B (**66**), were isolated from a specimen of *A. aureum*, which collected at depth more than 28 m at Sodwane Bay, South Africa. The two compounds showed a cytotoxic effect against P-388 mouse leukemia, A-549 human lung carcinoma, MEL-28 human melanoma, and HT-29 human colon carcinoma [44]. 

Pukalide (**67**) has been reported from soft coral *A. antarticum*, which was collected during the XVII Italian campaign in Antartica off Terra Nova Bay [32]. It is a known diterpene, which was previously reported from *Sinularia abrupta*. Pukalide showed feeding-deterrence against *Carassius auratus* at a concentration of 50 μg/mL [32,45].

### 2.3. Steroids

Gorgosterol (**68**) has been reported from *A. molle*, collected at Pioneer Bay, Orpheus Island. Its structure was elucidated based on ^I^H-NMR spectral data and other physical properties [40]. 

**Figure 10:**
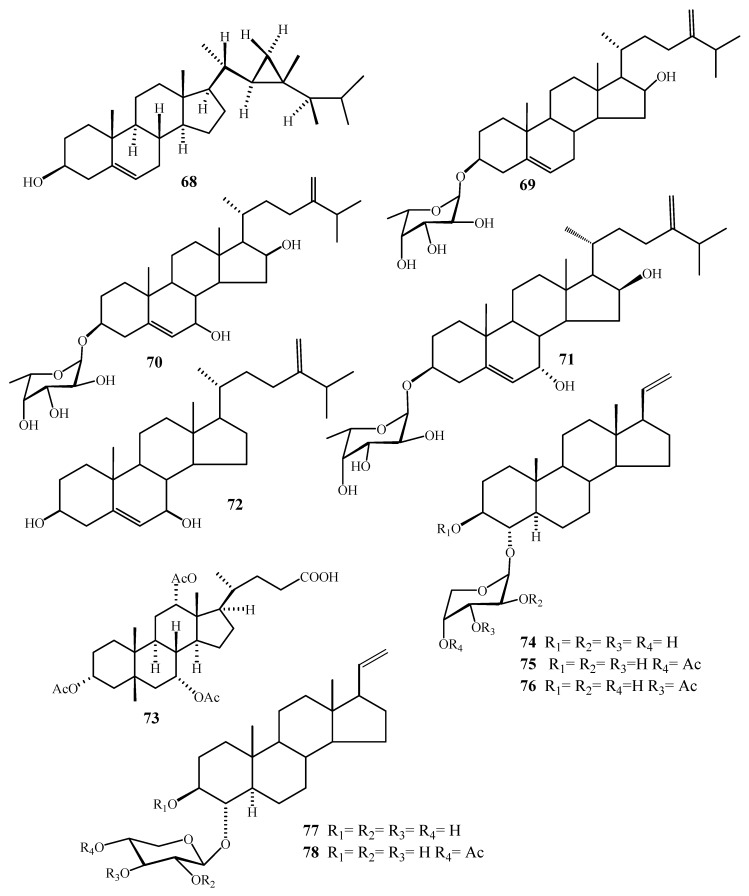
Chemical structures of compounds **68**–**78**.

Investigation of *Alcyonium* sp., which, was collected from the Andaman and Nicobar coasts, led to identification of three new polyhydroxysterol gyclosides, 24-methylenecholest-5-ene-3β, 16β-diol-3-*O*-α-l-fucoside (**69**) (Figure 10), 24-methylenecholest-5-ene-3β, 7β,16β-diol-3-*O*-α-l-fucopyranoside (**70**), and 24-methylenecholest-5-ene-3β,7α,16β-triol-3-*O*-α-l-fucopyranoside (**71**), along with the already reported polyhydroxy sterol 3β,7β-dihydroxy-24-methylenecholesterol (**72**). These compounds play an important role in the chemotaxonomical approach since they are rare in such soft coral [46]. 

A soft coral, *Alcyonium* sp., which was collected from the coast of southern Taiwan and found to produce 3α,7α,12α-triacetoxy-5β-cholanic acid (**73**). Its structure was assigned on the basis of spectroscopical data and its configuration was further supported by molecular mechanics calculations [47]. 

The acetone extract of *Alcyonium* sp., which was collected from Taketomijima, Okinawa, yielded rare five steroidal glycosides of pregnene-type (Pregnedioside-A, **74**), 4′-*O*-acetyl-pregnedioside-A (**75**), 3′-*O*-acetyl-pregnedioside-A (**76**), pregnedioside-B (**77**) and 4′-*O*-acetyl-pregnedioside (**78**). This was the first report of these steroidal compounds reported as glyco-conjugates from marine organisms [48]. 

Four new steroid derivatives 3-methoxy-19-norpregna-1,3,5(10),20-tetraene (**79**), 3-(4-*O*-acetyl-6-deoxy-β-galactopyranosyloxy)-19-norpregna-1,3,5(10),20-tetraene (**80**), 22,23-dihydroxy cholesta-1,24-dien-3-one (**81**), and methyl 3-oxochola-1,4,22-trien-24-oate (**82**) were isolated from *A. gracillimum*, which was collected from the Gulf of Sagami, Japan. The new steroids (**79**–**82**) were lethal to cyprids of barnacle (*Balanus amphitrite*) larvae, at 100 µg/mL, albeit showed no inhibition of larval settlement of *B. amphitrite* at 50 µg/mL [49]. 

**Figure 11:**
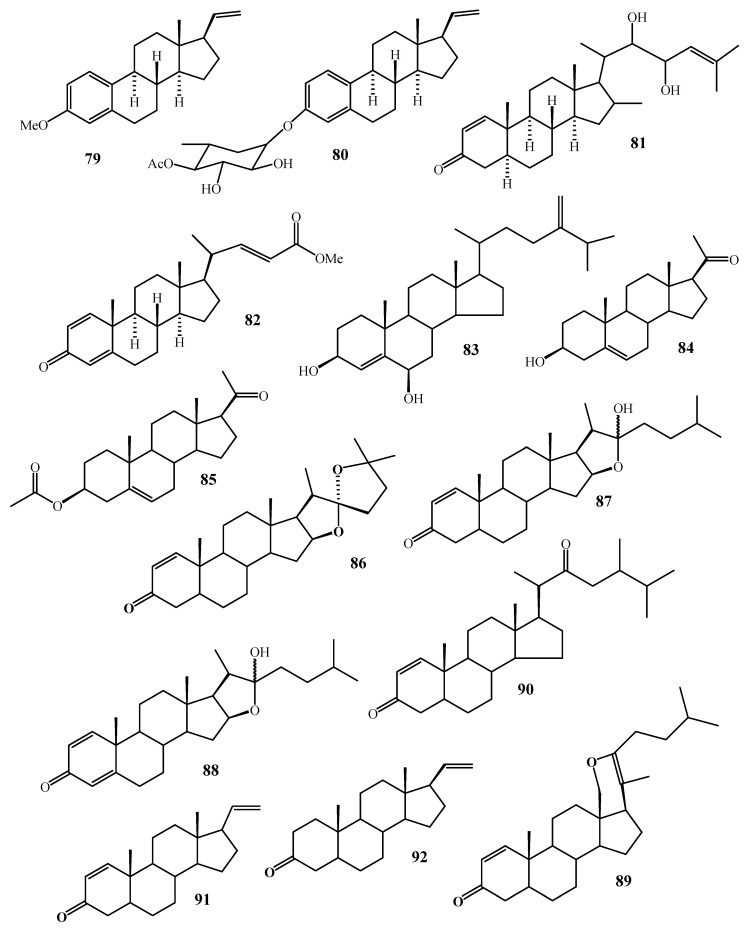
Chemical structures of compounds **79**–**92**.

A dihydroxy sterol, 24-methylenecholest 4-ene-3β,6β-diol (**83**) (Figure 11), has been isolated from *A. patagonicum*, which was collected from the south China Sea. It had cytotoxic against the P-388 cell line [32]. 

Two well-known steroids, pregnenolone (**84**) and pregnenolone-3-acetate (**85**), have been isolated from the soft coral, *A. antarticum*, which was collected during the XVII Italian campaign in Antartica off Terra Nova Bay [31]. Seven steroids, five of which, were new steroids; furospirostan class with spiroketal functionality (**86**), two steroids with hemiketal functionality (**87**–**88**), steroid with unusual dihydropyran ring (**89**) and a steroidal ketoic derivatives (**90**) have been reported for the first time from *A. gracillimum* and already two known steroids of pregnane class pregnadienone and pregnenone (**91**–**92**). Interestingly, the crude extract of *A. gracillimum* exhibited moderate cytotoxicity (IC_50_ 22.4 µg/mL) and antiviral activity (IC_50_ 7.8 µg/mL) against P388 and HSV-I, respectively. Compounds (**87**–**88**) exhibited moderate inhibition against human cytomegalovirus (IC_50_ 3.7 and 7.2 µg/mL, respectively) [50].

## 3. *Alcyonium* Terpenoids; Current State and Future Aspect

Terpenes are secondary metabolites, mainly derived from the five carbo-skeleton isoprene unit [51]. Derivatization or modifications of these units resulted in a diversity of molecular structures with unlimited chemical and biological characters. Up-to-date huge marine terpenoidal derivatives were reported from invertebrates, particularly, soft corals with interesting structures. Since discovering of marine terpenoids in the 1970s, several reviews devoted to describing the diversity of their chemical structures; monoterpenoids, diterpenoids, sesterterpenes, triterpenoid oligoglycosides and sterols [52,53,54,55,56,57,58,59,60,61,62,63,64,65,66,67,68,69,70,71]. These publications described the importance and features of chemically mediated interactions among marine organism and their role as a defense mechanism [61,62,63,64,65,66,67,68,69,70,71]. 

As aforementioned there are diversity of terpenoidal classes are presented; sesquiterpenoids (aphanmalane, aromadendrane, bulgarane, cadinane, bicyclogermacrane, eudesmane, furanosesquiterene, guaiazulene illudalane, paesslerane, and triprenylhydroquinone); diterpenoids (cembrane, cladiellin, eunicellin, prenylbicyclogermacrane, xenicin, and xenicane); and steroids (cholestane (C-27), campestane (C-28), gorgostane (C-30) along with pregnane (C21)). Unfortunately, the reported alcyonacean metabolites are still biologically unscreened. For instance, thirteen macrocyclic ‘cembranoid’ diterpenes (**43**–**54**, **67**) urgently require substantial examination. Other cembranoidal derivatives with similar features showed an important finger-print in terms of pharmacological applications, which embrace antimicrobial, anti-proliferative, and anti-inflammatory properties [72,73,74,75,76,77,78,79,80,81]

It is wealthy to highlight the fact, which is presented in Table 1, that eight out of 42 sesquiterpenes, 20 out of 25 diterpenes and 18 out of 25 steroids were biologically unscreened. This indicated that 50% of the isolated compounds from genus *Alcyonium* still require further examination. 

*Alcyonium* is considered as a potential source for nitrogenous and non-nitrogenous terpenoidal derivatives. By the way, *A. paessleri* produces rare nitrogen containing illudalane sesquiterpene (alcyopterosins B, C, E, F, G, H, J and M) [29]. Thus, this review focused on elaborating the future plan for the natural products researchers to investigate the disremembered genus *Alcyonium*. 

## 4. Conclusions

*Alcyonium* could be considered as a potential source of bioactive terpenoidal metabolites. The engagement of different approaches played a significant role in the facilitation of the forthcoming drug discovery process. Remarkable, many marine metabolites displaying fascinating molecular structures with diverse pharmacological effects have been reported from genus *Alcyonium* during the last four decades (1981–2019). Of the 92 distinctive structures accounted for in this review, 67 (72.8%) are terpenoidal metabolites. 

**Figure 12:**
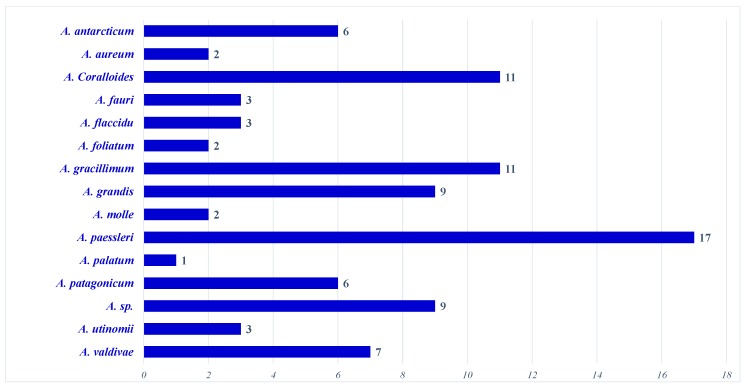
Number of compounds reported from *Alcyonium* species.

Figure 12 illustrates terpenoidal metabolites produced by 16 species. The majority (41.8%) of the presented compounds were produced by three species; *A. paessleri* (17 compounds, 18.5%), *A. coralloides* (12 compounds, 12.0%) and *A. gracillimum* (11 compounds, 12.0%), respectively.

## Figures and Tables

**Table 1:** Terpenoidal metabolites isolated from genus *Alcyonium*.

Cpd. No.	Cpd. Name	Species	Biological Effects	Class of Cpd	Ref. No.
**1**	Guaiazulene	*Alcyonium* sp.	-	Guaiazulene	[22]
**2**	(+)-Coralloidin-A	*A. coralloides*	-	Eudesmane sesquiterpene	[23,24]
**3**	(−)-Coralloidin- B		-	Bicyclogermacrane	
**4**–**6**	Coralloidin C, D and E		-	Eudesmane sesquiterpene	
**7**–**8**	Millecrone A and B	*A. foliatum* and *A. valdiviae*	Antifungal	Aphanmalane sesqui. Aromadendrane sesqui.	[25]
**9**–**10**	Millecrol A and B		Antimicrobial	Aphanmal sesqui. Cadinane sesqui.	[26]
**11**	Furanosesquiterpenoid	*A. palmatum*	Antifeedant	Furanosesquiterpene	[27]
**12**	Rietone	*A. fauri*	Anti-HIV	Triprenylhydroquinone	[28]
**13**	8′-Acetoxyrietoneand				
**14**	8′-Desoxyrietone				
**15**–**29**	Alcyopterosins A-O	*A. paessleri*	Cytotoxic	Illudalane Sesquiterpene	[29]
**30**–**31**	Paesslerins A and B	*A. paessleri*	-	Paesslerane sesquiterpene	[30]
**32**	4,12-Bis-*n*-butanoylalcyopterosin O,	*A. grandis*	Antifeedant	Illudalane Sesquiterpene	[31]
**33**	13-Acetoxy-12-acetylalcyopterosin D				
**34**	4,12-Bis(acetyl) alcyopterosin O				
**35**	12-Acetyl-13-*n*-butanoxyalcyopterosin D				
**36**	12-Acetyl-4-*n*-butanoylalcyopterosin O				
**37**	12-Acetylalcyopterosin D				
**38**	12-*n*-Butanoylalcyopterosin D				
**39**	13-Hydroxy alcyopterosin and				
**40**	Alcyopterosin P				
**41**	Alcyonicene	*A. antarcticum*	Feeding-deterrence and ichthyotoxic	Bulgarane sesquiterpene	[32]
**42**	Deacetoxyalcyonicene		-		
**43**	Flaccidoxide	*A. flaccidum*	-	Cembrane diterpene	[33]
**44**	Cembrene-C				
**45**	Sarcophytol B				
**46**	Alcyonol-A	*A. utinomii*			[34]
**47**	Alcyonol-B				
**48**	Alcyonol-C				
**49**–**54**	Coralloidolide (A–F)	*A. coralloides*		Cembrane diterpene	[35,36,37]
**55**	Alcyonolide	*Alcyonium* sp.		Xenicin diterpene	[38]
**56**	Alcyonolide-5				[39]
**57**	(l*S*,2*R*,3*S*,4*R*,5*R*,6*S*,8*E*,ll*S*,l2*R*,13*S*,14*S*)-3-Acetoxy-2,12-dibutanoyloxycladiell-8-ene-4,Il-diol	*A. molle*		Cladiellin diterpene	[40]
**58**	Patagonicol	A. *patagonicum*		Eunicellin diterpene	[41]
**59**	Valdivone A	*A. valdivae*	Anti-inflammatory	Eunicellin diterpene	[42]
**60**	Valdivone B				
**61**	4-*O*-Methyl valdivone A		-		
**62**	4-*O*-Methyl valdivone B		-		
**63**	Dihydrovaldivone A				
**64**	Palmatol	*A. palmatum*		Prenylbicyclogermacrane	[43]
**65**–**66**	Zahavin A, and zahavin B	*A. aureum*	Cytotoxic	Xenicane diterpene	[44]
**67**	Pukalide	*A. antarticum*	Feeding-deterrence	Cembrane diterpene	[45]
**68**	Gorgosterol	*A. molle*	-	Gorgosterol	[40]
**69**	24-Methylenecholest-5-ene-3β,16β-diol-3-*O*-α-l-fucoside	*Alcyonium* sp.		Campestane	[46]
**70**	24-Methylenecholest-5-ene-3β,7β,16β-triol-3-*O*-α-l-fucopyranoside				
**71**	24-Methylenecholest-5-ene-3β,7α,16β-triol-3-*O*-α-l-fucopyranoside				
**72**	3β,7β-Dihydroxy-24-methylenecholesterol				
**73**	3α,7α,12α-Triacetoxy-5β-cholanic acid			Cholestane	[47]
**74**	Pregnedioside-A	*Alcyonium* sp.		Pregnane	[48]
**75**	4′-*O*-Acetyl-pregnedioside-A				
**76**	3′-*O*-Acetyl-pregnedioside-A				
**77**	Pregnedioside-B				
**78**	4′-*O*-Acetyl-pregnedioside				
**79**	3-Methoxy-19-norpregna-1,3,5(10),20-tetraene	*A. gracillimum*	Antifoulants	Pregnane	[49]
**80**	3-(4-*O*-Acetyl-6-deoxy-β-galactopyranosyloxy)-19-nor-pregna-1,3,5(10),20-tetraene				
**81**	22,23-Dihydroxycholesta-1,24-dien-3-one			Cholestane	
**82**	methyl Methyl-3-oxochola-1,4,22-trien-24-oate				
**83**	24-Methylenecholest 4-ene-3β,6β-diol	*A. patagonicum*	Cytotoxic	Campestane	[41]
**84**	Pregnenolone	*A. antarticum*	-	Pregnane	[32]
**85**	Pregnenolone-3-acetate				
**86**	Furospirostan	*A. gracillimum*		Cholestane	[50]
**87**–**88**	Cholestane derivative with hemiketal functionality		Cytotoxic		
**89**	Steroid with unusual dihydropyran ring		-		
**90**	Ketosteroidal derivatives				
**91**	Pregnadienone			Pregnane	
**92**	Pregnenone		-		

## References

[1] Newman D.J., Cragg G.M. (2016). Drugs and Drug Candidates from Marine Sources: An Assessment of the Current “state of Play”. Planta Med..

[2] Arrieta J.M., Arnaud-Haond S., Duarte C.M., Gaines S.D. (2010). What lies underneath: Conserving the oceans’ genetic resources. Proc. Natl. Acad. Sci. USA.

[3] McFadden C.S., Ofwegen L.P. (2017). VAN Revisionary systematics of the endemic soft coral fauna (Octocorallia: Alcyonacea: Alcyoniina) of the Agulhas Bioregion, South Africa. Zootaxa.

[4] WoRMS-World Register of Marine Species. http://www.marinespecies.org.

[5] Gage J.D., Tyler P.A. (1992). Deep-Sea Biology, A Natural History of Organisms at the Deep-Sea Floor.

[6] Shang J., Hu B., Wang J., Zhu F., Kang Y., Li D., Sun H., Kong D.X., Hou T. (2018). Cheminformatic Insight into the Differences between Terrestrial and Marine Originated Natural Products. J. Chem. Inf. Model..

[7] Blunt J.W., Copp B.R., Keyzers R.A., Munro M.H.G., Prinsep M.R. (2017). Marine natural products. Nat. Prod. Rep..

[8] Núñez-Pons L., Carbone M., Vázquez J., Gavagnin M., Avila C. (2013). Lipophilic defenses from *Alcyonium* soft corals of Antarctica. J. Chem. Ecol..

[9] Hegazy M.E.F., Mohamed T.A., Alhammady M.A., Shaheen A.M., Reda E.H., Elshamy A.I., Aziz M., Paré P.W. (2015). Molecular architecture and biomedical leads of terpenes from Red Sea marine invertebrates. Mar. Drugs.

[10] Choudhary A., Naughton L.M., Mont I., Dobson A.D.W., Rai D.K. (2017). Current Status and Future Prospects of Marine Natural Products (MNPs) as Antimicrobials. Mar. Drugs.

[11] Kong D.X., Jiang Y.Y., Zhang H.Y. (2010). Marine natural products as sources of novel scaffolds: Achievement and concern. Drug Discov. Today.

[12] Tripathi V.C., Satish S., Horam S., Raj S., lal A., Arockiaraj J., Pasupuleti M., Dikshit D.K. (2018). Natural products from polar organisms: Structural diversity, bioactivities and potential pharmaceutical applications. Polar Sci..

[13] Fine M., Cinar M., Voolstra C.R., Safa A., Rinkevich B., Laffoley D., Hilmi N., Allemand D. (2019). Coral reefs of the Red Sea-challenges and potential solutions. Reg. Stud. Mar. Sci..

[14] Alarif W.M., Abdel-Lateff A., Alorfi H.S., Alburae N.A. (2019). Alcyonacea: A Potential Source for Production of Nitrogen-Containing Metabolites. Molecules.

[15] Chanmethakul T., Chansang H., Watanasit S. (2010). Soft coral (Cnidaria: Alcyonacea) distribution patterns in Thai waters. Zool. Stud..

[16] Mayer A.M.S., Glaser K.B., Cuevas C., Jacobs R.S., Kem W., Little R.D., McIntosh J.M., Newman D.J., Potts B.C., Shuster D.E. (2010). The odyssey of marine pharmaceuticals: A current pipeline perspective. Trends Pharmacol. Sci..

[17] König G.M., Kehraus S., Seibert S.F., Abdel-Lateff A., Müller D. (2006). Natural products from marine organisms and their associated microbes. ChemBioChem.

[18] Faulkner D.J. (2000). Marine pharmacology. Antonie van Leeuwenhoek. Int. J. Gen. Mol. Microbiol..

[19] Newman D.J., Cragg G.M. (2017). Current Status of Marine-Derived Compounds as Warheads in Anti-Tumor Drug Candidates. Mar. Drugs.

[20] McFadden C., van Ofwegen L. (2013). Molecular phylogenetic evidence supports a new family of octocorals and a new genus of Alcyoniidae (Octocorallia, Alcyonacea). Zookeys.

[21] Mcfadden C.S., Donahue R., Hadland B.K., Weston R. (2001). A molecular phylogenetic analysis of reproductive trait evolution in the soft coral genus alcyonium published by: The society for the study of evolution a molecular phylogenetic analysis of reproductive trait evolution in the soft coral genus alcyonium. Evolution.

[22] Bowden B.B.F., Coll J.J.C., Tapiolas D.M.D. (1983). Studies of Australian Soft Corals. XXX A Novel Trisnorsesquiterpene from a Cespitularia Species and the Isolation of Guaiazulene from a Small Blue *Alcyonium* Species. Aust. J. Chem..

[23] Guerriero A., Dematté B., D’Ambrosio M., Pietra F. (1986). (+)-Coralloidin-A and (−)-Coralloidin-B, Two New Sesquiterpenoids from the Mediterranean Alcyonacean *Alcyonium coralloides*. J. Nat. Prod..

[24] D’Ambrosio M., Guerriero A., Pietra F. (1987). Coralloidin C, D, and E: Novel Eudesmane Sesquiterpenoids from the Mediterranean Alcyonacean *Alcyonium coralloides*. Helv. Chim. Acta.

[25] Pika J., Faulkner D.J. (1994). Four sesquiterpenes from the South African nudibranch *Leminda millecra*. Tetrahedron. J. Nat. Prod..

[26] McPhail K.L., Davies-Coleman M.T., Starmer J. (2001). Sequestered chemistry of the Arminacean nudibranch *Leminda millecra* in Algoa Bay, South Africa. J. Nat. Prod..

[27] Cimino G., De Rosa S., De Stefano S., Sodano G. (1984). A new furanosesquiterpene from the Mediterranean Alcyonacean *Alcyonum Palmatum*. J. Nat. Prod..

[28] Hooper G.J., Davies-Coleman M.T. (1995). Sesquiterpene hydroquinones from the South African soft coral *Alcyonium fauri*. Tetrahedron Lett..

[29] Palermo J.A., Brasco M.F., Spagnuolo C., Seldes A.M. (2000). Illudalane sesquiterpenoids from the soft coral *Alcyonium paessleri*: The first natural nitrate esters. J. Org. Chem..

[30] Rodríguez Brasco M.F., Seldes A.M., Palermo J.A. (2001). Paesslerins A and B: Novel Tricyclic Sesquiterpenoids from the Soft Coral *Alcyonium Paessleri*. Org. Lett..

[31] Carbone M., Núñez-Pons L., Castelluccio F., Avila C., Gavagnin M. (2009). Illudalane Sesquiterpenoids of the Alcyopterosin Series from the Antarctic Marine Soft Coral *Alcyonium grandis*. J. Nat. Prod..

[32] Manzo E., Ciavatta M.L., Nuzzo G., Gavagnin M. (2009). Terpenoid content of the Antarctic soft coral *Alcyonium antarcticum*. Nat. Prod. Commun..

[33] Kashman Y., Carmely S., Groweiss A. (1981). Further Cembranoid Derivatives from the Red Sea Soft Corals *Alcyonium flaccidum* and *Lobophytum crassum*. J. Org. Chem..

[34] Kinamoni Z., Groweiss A., Carmely S., Kashman Y., Loya Y. (1983). Several new cembranoid diterpenes from three soft corals of the red sea. Tetrahedron.

[35] Ambrosio M.D., Fabbri D., Guerriero A., Pietra F., Chimica I., Trento U. (1986). Coralloidolide A and Coralloidolide B, the First Cembranoids from a Mediterranean Organism, the Alcyonacean *Alcyonium coralloides*. Helv. Chim. Acta.

[36] D’Ambrosio M., Guerriero A., Pietra F. (1989). Novel cembranolides (Coralloidolide D and E) and a 3,7-Cyclized cembranolide (Coralloidolide C) from the mediterranean coral *Alcyonium coralloides*. Helv. Chim. Acta.

[37] D’Ambrosio M., Guerriero A., Pietra F. (1990). Coralloidolide F, the First Example of a 2,6-Cyclized Cembranolide: Isolation from the Mediterranean Alcyonacean Coral *Alcyonium coralloides*. Helv. Chim. Acta.

[38] Kobayashi M., Yasuzawa T., Kobayashi Y., Kyogoku Y., Kitagawa I. (1981). Alcyonolide, a novel diterpenoid from a soft coral. Tetrahedron Lett..

[39] Coll J.C., Kearns P.S., Rideout J.A. (1998). Isolation of a novel diterpene triacetate from two soft corals of the order Alcyonacea. J. Nat. Prod..

[40] Bowden B.F., Coll J.C., Dai M.C. (1989). Studies of australian soft corals. XLIII the structure elucidation of a new diterpene from *Alcyonium molle*. Aust. J. Chem..

[41] Su J., Zheng Y., Zeng L., Pordesimo E.O., Schmitz F.J., Hossain M.B., van der Helm D. (1993). Patagonicol: A diterpenoid from the Chinese soft coral *Alcyonium patagonicum*. J. Nat. Prod..

[42] Lin Y., Bewley C.A., Faulkner D.J.J. (1993). The valdivones, anti-inflammatory diterpene esters from the South African soft coral *Alcyonium valdivae*. Tetrahedron.

[43] Zubia E., Spinella A., Giusto G.B., Crispinoand A., Cimino G. (1994). A new diterpenoid skeleton from the Mediterranean octocoral *Alcyonium palmatum*: Structure of palmatol. Tetrahedron Lett..

[44] Rudi A., Ketzinel S., Goldberg I., Stein Z., Kashman Y., Benayahu Y., Schleyer M. (1995). Antheliatin and Zahavins A and B, three new cytotoxic xenicane diterpenes from two soft corals. J. Nat. Prod..

[45] Missakian M.G., Burreson B.J., Scheuer P.J. (1975). Pukalide, a furanocembranolide from the soft coral *Sinularia abrupta*. Tetrahedron.

[46] Kobayashi M., Kanda F., Damarla S.R., Rao D.V., Rao C.B. (1990). Marine sterols. XVII. Polyhydroxysterols of the soft corals of the andaman and nicobar coasts. (2). Isolation and atructures of three 16β-hydroxy steroidal glycosides from an *Alcyonium* sp. soft coral. Chem. Pharm. Bull..

[47] Chen W.-C., Sheuz J.-H., Fangy L.-S., Hux W.-P., Sung P.-J. (2006). 3α,7α,12α-Triacetoxy-5β-cholanic acid, a steroid from the Formosan soft coral *Alcyonium* sp. (Alcyoniidae). Nat. Prod. Res..

[48] Kobayashi M., Kiyota Y., Orito S., Kyogoku Y., Kitagawa I. (1984). Five new steroidal glycosides, pregnedioside-A, -B, and their three monoacetates, from an Okinawan soft coral of *Alcyonium* sp.. Tetrahedron Lett..

[49] Tomono Y., Hirota H., Imahara Y., Fusetani N. (1999). Four new steroids from two octocorals. J. Nat. Prod..

[50] Seo Y., Jung J.H., Rho J.-R., Shin J., Song J.-I. (1995). Isolation of novel bioactive steroids from the soft coral *Alcyonium gracillimum*. Tetrahedron.

[51] Ruzicka L. (1953). The isoprene rule and the biogenesis of terpenic compounds. Experientia.

[52] Blunt J.W., Copp B.R., Munro M.H.G., Northcote P.T., Prinsep M.R. (2005). Marine natural products. Nat. Prod. Rep..

[53] Fraga B.M. (2005). Natural sesquiterpenoids. Nat. Prod. Rep..

[54] Hanson J.R. (1996). The sesterterpenoids. Nat. Prod. Rep..

[55] Coll J.C., Bowden B.F., Tapiolas D.M., Willis R.H., Djura P., Streamer M., Trott L. (1985). The terpenoid chemistry of soft corals and its implications. Tetrahedron.

[56] Hay M.E., Fenical W. (1988). Marine plant–herbivore interactions: The ecology of chemical defense. Ann. Rev. Ecol. Syst..

[57] Minale L., Iorizzi M., Palagiano E., Riccio R. (1996). Steroid and triterpenoid oligogylcosides of marine origin. Adv. Exp. Med. Biol..

[58] Djerassi C. (1981). Recent studies in the marine sterol field. Pure Appl. Chem..

[59] Hanson J.R. (2005). Diterpenoids. Nat. Prod. Rep..

[60] Pennock J.F. (1977). Terpenoids in marine invertebrates. Int. Rev. Biochem..

[61] Andersen R.J., De Silva E.D., Dumdei E.J., Northcote P.T., Pathirana C., Tischler M., Towers G.H.N., Stafford H.A. (1990). Terpenoids from selected marine invertebrates. Recent Advances in Phytochemistry.

[62] Bakus G.J., Targett N.M., Schulte B. (1986). Chemical ecology of marine organisms: An overview. J. Chem. Ecol..

[63] Hay M.E. (1996). Marine chemical ecology: What’s known and what’s next?. J. Exp. Mar. Biol. Ecol..

[64] Connolly J.D., Hill R.A. (2005). Triterpenoids. Nat. Prod. Rep..

[65] Paul V.J., Puglisi M.P. (2004). Chemical mediation of interactions among marine organisms. Nat. Prod. Rep..

[66] Coll J.C. (1992). The chemistry and chemical ecology of octocorals (Coelenterata, Anthozoa, Octocorallia). Chem. Rev..

[67] Fusetani N. (2004). Biofouling and antifouling. Nat. Prod. Rep..

[68] McClintock J.B., Baker B.J. (1997). A review of the chemical ecology of Antarctic marine invertebrates. Am. Zool..

[69] Proksch P. (1994). Defensive roles for secondary metabolites from marine sponges and sponge-feeding nudibranchs. Toxicon.

[70] Zubair M., Alarif W.M., Al-Footy K.O., PH M., Aly M., Basaif S., Al-Lihaibi S., Ayyad S.-E. (2016). New antimicrobial biscembrane hydrocarbon and cembranoid diterpenes from the soft coral Sarcophyton trocheliophorum. Turk. J. Chem..

[71] Welford A.J., Collins I. (2011). The 2,11-cyclized cembranoids: Cladiellins, asbestinins, and briarellins (period 1998–2010). J. Nat. Prod..

[72] Hsiao T.-H., Sung C.-S., Lan Y.-H., Wang Y.-C., Lu M.-C., Wen Z.-H., Wu Y.-C., Sung P.-J. (2015). New Anti-Inflammatory Cembranes from the Cultured Soft Coral *Nephthea columnaris*. Mar. Drugs.

[73] Hsiao T.-H., Cheng C.-H., Wu T.-Y., Lu M.-C., Chen W.-F., Wen Z.-H., Dai C.-F., Wu Y.-C., Sung P.-J. (2015). New Cembranoid Diterpenes from the Cultured Octocoral Nephthea columnaris. Molecules.

[74] Huang H.-W., Tang J.-Y., Ou-Yang F., Wang H.-R., Guan P.-Y., Huang C.-Y., Chen C.-Y., Hou M.-F., Sheu J.-H., Chang H.-W. (2018). Sinularin Selectively Kills Breast Cancer Cells Showing G2/M Arrest, Apoptosis, and Oxidative DNA Damage. Molecules.

[75] Noah C.-A., Wu W.-T., Dai G.-F., Su J.-H., Liu C.-I., Su T.-R., Wu Y.-J. (2018). Flaccidoxide-13-Acetate Extracted from the Soft Coral Cladiella kashmani Reduces Human Bladder Cancer Cell Migration and Invasion through Reducing Activation of the FAK/PI3K/AKT/mTOR Signaling Pathway. Molecules.

[76] Lee Y.-S., Duh T.-H., Siao S.-S., Chang R.-C., Wang S.-K., Duh C.-Y. (2017). New Cytotoxic Terpenoids from Soft Corals *Nephthea chabroli* and *Paralemnalia thyrsoides*. Mar. Drugs.

[77] Wu J., Xi Y., Huang L., Li G., Mao Q., Fang C., Shan T., Jiang W., Zhao M., He W. (2018). A Steroid-Type Antioxidant Targeting the Keap1/Nrf2/ARE Signaling Pathway from the Soft Coral *Dendronephthya gigantea*. J. Nat. Prod..

[78] Wu Q., Li H., Yang M., Jia A.-Q., Guo Y.-W. (2019). Two new cembrane-type diterpenoids from the xisha soft coral Lemnalia flava. Fitoterapia.

[79] Zhang Q., Liang L.-F., Miao Z.-H., Wu B., Guo Y.-W. (2019). Cytotoxic polyhydroxylated steroids from the South China Sea soft coral *Lobophytum* sp.. Steroids.

[80] Zhang Q., Li X.-W., Yao L.-G., Wu B., Guo Y.-W. (2019). Three new capnosane-type diterpenoids from the South China Sea soft coral *Lobophytum* sp.. Fitoterapia.

[81] Wu Q., Li X.-W., Li H., Yao L.-G., Tang W., Miao Z.-H., Wang H., Guo Y.-W. (2019). Bioactive polyoxygenated cembranoids from a novel Hainan chemotype of the soft coral *Sinularia flexibilis*. Bioorg. Med. Chem. Lett..

